# Generating Alzheimer’s narratives using large language models

**DOI:** 10.1186/s12911-026-03653-4

**Published:** 2026-07-07

**Authors:** Paula Andrea Perez-Toro, Mahmoud Almizel, Elmar Nöth, Andreas Maier, Tomas Arias-Vergara

**Affiliations:** 1 Pattern Recognition Lab, Friedrich-Alexander-Universität Erlangen-Nürnberg, Erlangen, Germany; 2 GITA Lab, University of Antioquia, Medellín, Colombia

**Keywords:** Large language models, Alzheimer’s disease, Synthetic data augmentation, Generative AI

## Abstract

**Background:**

Analyzing semi-spontaneous speech is a promising direction for supporting Alzheimer’s disease (AD) assessment, yet progress is limited by the scarcity of annotated clinical data. Large Language Models (LLMs) offer new opportunities to generate synthetic narratives that may resemble speech patterns of both patients with AD and healthy controls during cognitive evaluation tasks such as the Cookie Theft Picture description.

**Methods:**

This study evaluates whether models including GPT, T5/Flan-T5, LLaMA, Mistral, and Qwen can generate clinically plausible picture-description narratives under two configurations: Human-to-Bot, where an LLM responds directly to real interviewer prompts, and Bot-to-Bot, where two LLMs simulate both interviewer and participant roles. Models were fine-tuned on transcripts from the DementiaBank Pitt Corpus and assessed using lexical and semantic metrics, as well as human expert ratings. Generated narratives were further used to augment training data for an AD vs. healthy control classifier based on BERT embeddings and an MLP architecture.

**Results:**

LLMs differed substantially in their ability to reproduce clinically meaningful and semantically coherent narratives of patient-interviewer interactions. Mistral, LLaMA, and Qwen achieved the strongest automatic evaluation metrics, e.g., BERTScores above 0.90 in the Human-to-Bot condition—and produced narratives rated by human experts as fluent, plausible, and diagnostically informative. When combining real and synthetic narratives for classifier training, the highest F1-score reached 0.84, outperforming models trained on real data alone (F1 = 0.74). Synthetic data generated in Human-to-Bot settings contributed most to diagnostic improvements, whereas Bot-to-Bot interactions exhibited greater variability and reduced clinical realism.

**Conclusion:**

LLMs can generate high-quality synthetic narratives that enhance downstream AD classification and show promising clinical plausibility in cognitive assessment contexts. Incorporating LLM-generated data provides a scalable strategy for mitigating data scarcity in dementia research. Future work should focus on improving fully synthetic dialogue quality, expanding multilingual capabilities, and refining evaluation frameworks to better capture clinically relevant linguistic features.

**Supplementary Information:**

The online version contains supplementary material available at 10.1186/s12911-026-03653-4.

## Introduction

Language models, particularly those using Question-Answering (QA) capabilities, have demonstrated significant utility in simulating dynamic interactions that mimic the complexities of human conversation [1–5]. Expanding on these approaches, this paper explores the integration of Large Languages Models (LLMs) to generate synthetic interviews during cognitive assessments in Alzheimer’s Disease (AD). In this context, synthetic data refers to artificially generated narrative responses produced by LLMs that aim to approximate the linguistic and interactional characteristics of real patient speech. Such data can support research by mitigating clinical data scarcity and enabling controlled data augmentation.

AD is the most common type of dementia characterized by progressive degeneration of neurons in the cerebral cortex and hippocampus, which is caused by brain tissue changes and a loss of a chemical vital to brain function called acetylcholine [6]. Consequently, AD leads to memory loss, psychological changes, and cognitive impairments [7]. These changes can disrupt speech fluency, hinder access to semantic information, and cause abnormalities in language production [7].

Picture description tasks are widely used in neuropsychological assessment, particularly for early cognitive screening and differential diagnosis. The Cookie Theft Picture [8], from the Boston Diagnostic Aphasia Examination [9], is a standard tool for assessing cognitive and language impairments in AD. In this task, individuals are asked to describe a scene depicting a family in a kitchen, detailing events and objects, while the examiner may provide prompts to elicit more comprehensive descriptions. The task engages cognitive processes such as attention, visual perception, memory, and language production, offering insights into speech clarity, vocabulary use, grammatical structure, and discourse organization. Clinicians analyze linguistic features including lexical retrieval difficulties, semantic substitutions, reduced syntactic complexity, disfluencies, and decreased informativeness. These markers contribute to diagnostic reasoning and longitudinal monitoring of cognitive decline. The present work does not aim to replace clinical evaluation, but rather to support computational tools that may assist large-scale screening efforts and research-oriented assessment workflows.

### Related work

LLMs have shown strong potential in Alzheimer’s Disease (AD) detection, particularly through the analysis of spontaneous speech and integration of multimodal data [10]. Models such as GPT have been used to identify early cognitive impairments by detecting features such as word-finding issues and grammatical errors [11]. Besides using as assistive tools, several studies have adopted NLP techniques to automatically detect AD [12–17].

Much of this work relies on the Pitt Corpus from DementiaBank [18], a dataset comprising spontaneous speech from native American English speakers. The ADReSS and ADReSSo challenge datasets [19], which are curated subsets of this corpus, have served as standard benchmarks for AD detection. Results are typically reported on held-out test sets. For instance, Balagopalan et al. [20] demonstrated that fine-tuned Bidirectional Encoder Representations from Transformers (BERT) [21] models could achieve up to 83% accuracy in AD classification, compared to approximately 77% without fine-tuning.

In addition to detection tasks, LLMs also extend support to caregivers, particularly in managing AD care. Systems such as ADQueryAid [1] offers personalized assistance and improving usability in care settings [1]. This tool helps reduce the burden on caregivers by delivering accurate and necessary support promptly. Previous work has shown the suitability of LLMs to generate synthetic data from AD patients by identifying different categories of AD signs from unstructured clinical notes [22].

Another promising direction involves using LLMs for data augmentation. Generating synthetic interview transcripts and related content has been shown to enhance training datasets [23]. The creation of synthetic data combats the challenge of data scarcity, facilitating the development of richer and more diverse datasets [5]. This approach is exemplified by the production of synthetic interview transcripts, which have been proven effective in training models to identify conditions like PTSD and depression [24].

Finally, LLMs have found application in interactive diagnostic settings. Tools such as the State-Aware Patient Simulator (SAPS) simulate patient-doctor conversations, enabling scalable evaluation of clinical models [3]. Related techniques have been explored in other domains, such as followQG, which improves candidate screening by generating follow-up questions in video interviews [2].

### Contributions of this paper

Motivated by recent advances, this paper investigates the use of LLMs to generate synthetic interviews for cognitive assessments in AD, aiming to enrich training data for diagnostic models. Our key contributions are:

- This study explores the use LLMs to generate synthetic interviews for cognitive assessments in AD, to support data augmentation in AD diagnostics.
- We present a methodology to evaluate these models across diverse interaction scenarios, including Human-to-Bot and Bot-to-Bot, offering insights into their adaptability to variations in human input.
- We demonstrate that integrating synthetic narratives can enhance diagnostic accuracy and generalizability in distinguishing AD patients from Healthy Controls (HC).

## Materials & methods

### Data

The DementiaBank Pitt Corpus [18] is a widely used benchmark dataset for analyzing speech and language impairments in Alzheimer’s disease (AD). It contains audio recordings and manually transcribed interviews from individuals with AD and healthy control (HC) subjects, all native English speakers from the United States. In total, the corpus includes 552 interviews, comprising 309 from AD participants and 243 from HC participants. The recordings were primarily collected using the *Cookie Theft Picture* description task [8], a standard elicitation paradigm for assessing language production in dementia research. The corpus is publicly available and has been extensively used in studies on dementia-related language analysis. Table 1 presents the demographic characteristics of the participants at the subject level. Statistics are computed at the participant level to avoid multiple counting of longitudinal recordings.

**Table 1:** Demographic characteristics of participants with Alzheimer’s disease (AD) and healthy controls (HC). Values for age, Education, and MMSE are reported as mean (standard deviation) separately for females (F) and males (M)

	AD Patients	HC Subjects
Gender [F/M]	126 / 68	58 / 41
Age [F/M]	72.0 (8.6) / 69.2 (8.4)	63.3 (8.0) / 64.3 (7.9)
Education [F/M]	11.8 (2.6) / 13.0 (3.3)	14.0 (2.5) / 13.8 (2.5)
MMSE [F/M]	19.7 (4.9) / 21.0 (5.6)	29.2 (1.1) / 28.9 (1.1)

Narrative generation in these interviews begins with the Interviewer (INV) prompting the Participant (PAR) to describe the *Cookie Theft Picture* [8], followed by additional probing questions to elicit more detail when necessary.

Table 2 presents further details on the interaction frequency. The statistics detail the average number of sentences and words per sentence, along with the number of *interactions* and average words per *interaction* for each group between the participants and the interviewers. The statistics detail the average number of sentences and words per sentence, along with the number of *interactions* and average words per *interaction* for each group. In total, the corpus comprises 1,593 interactions, 1,102 from AD and 491 from HC. In this context, *interaction* encompasses the INV’s question and the PAR’s response. The corpus contains 9,606 sentences in total. These interactions span 9,606 sentences, with 5,683 sentences from AD patients and 3,923 from HC subjects, indicating that multiple sentences may occur within a single interaction.

**Table 2:** Statistical overview of interaction data between the participant (PAR) and the interviewer (INV). All values are expressed as average counts. AD: Alzheimer’s disease. HC: healthy controls

Group	Sentences	Words per Sentence	Interaction	Words per Interaction
**AD Patients**				
PAR	12.65	7.38	3.57	26.23
INV	5.74	4.08		7.02
**HC Subjects**				
PAR	12.94	8.07	2.0	51.82
INV	3.20	3.26		6.65

### Language models

We employed several state-of-the-art and open-source LLMs to analyze and mimic the narrative descriptions from the participants. Specifically, we used GPT-2, T5, Flan-T5, LLaMA, Mistral, and Qwen. Table 3 presents a comparative overview of the language models used, detailing their parameters, training prompt structures, and specific versions employed in this research.

**Table 3:** Comparison of language models used for QA in this paper. Params: parameters

Model	Size	Params	Prompt Structure
GPT-2	Small	124 M	Question:{} Answer:{}
	Medium	355 M	
	Large	774 M	
T5	Small	60 M	Answer this question:{}
	Base	220 M	
	Large	770 M	
LLaMA	7B	7B	<|im_start|>user {} <|im_end|> <|im_start|>assistant {} <|im_end|>
Mistral	Base	7B	
Qwen	8B	8B	

**Text-To-Text Transfer Transformer (T5)** is an encoder-decoder model [25], pre-trained on a mix of unsupervised and supervised tasks framed as text-to-text [26]. It employs a unified model architecture with both encoder and decoder elements of the Transformer [25], trained using a modified Masked Language Models (MLM) strategy called “span-corruption”, where contiguous random spans of text are replaced with a single mask token. It performs well on various tasks right out of the box by adding a specific prefix to the input for each task. We fine-tuned the T5 and Flan-T5 versions. Flan-T5 has the same architecture as T5; however, the difference lies in their training methodology, where Flan-T5 is fine-tuned on instruction-tuning [27]. We fine-tuned the small, base, and large versions of T5/Flan-T5.

**Generative Pre-trained Transformer 2 (GPT-2)** employs a Transformer-based architecture that exclusively uses the decoder component, focusing on next-word prediction to generate text [28]. In contrast to T5, GPT-2 does not require task-specific instructions to generate text. It uses a modified version of MLM without explicit masking; instead, the model applies Causal Language Modeling (CLM), which predicts all tokens of the input sequence sequentially. We fine-tuned the small, medium, and large GPT-2 checkpoints to generate patient/control narratives.

**Large Language Model–Meta AI (LLaMA)** is a high-performance language model developed by Meta AI, focusing on efficiency and scalability [29]. Unlike typical Transformer-based models that utilize extensive parameters, LLaMA optimizes both parameter use and computational resources, making it adept at handling a variety of NLP tasks with fewer resources. LLaMA is trained on a mixture of licensed data, publicly available data and data created by Meta AI, covering a wide range of languages and domains. LLaMA-2 models are pretrained on a mixture of publicly available and licensed data and further aligned using techniques such as supervised fine-tuning and Reinforcement Learning from Human Feedback (RLHF). The model’s ability to perform with limited training examples makes it particularly suitable for research and applications where data availability is constrained, such as ours. We fine-tuned the meta-llama/Llama-2-7b-hf^1^ checkpoint.

**Mistral AI** uses a hybrid training approach that combines supervised learning with RLHF [30]. This methodology allows Mistral to adapt to varied data distributions effectively and align its outputs more closely with human judgments and ethical standards. The model is based on the Transformer architecture with modifications for efficiency and scalability, trained initially on a large and diverse corpus followed by fine-tuning using RLHF to improve alignment with human preferences and ethical considerations. We fine-tuned the unsloth/mistral-7b-bnb-4bit checkpoint^2^.

**Qwen3 – Alibaba Cloud** is a LLM designed for multilingual understanding, reasoning, and instruction-following capabilities [31]. It uses the Transformer architecture and includes both dense and Mixture-of-Expert (MoE) variant sand integrates hybrid “thinking” and “non-thinking” modes, allowing the model to adaptively balance deep reasoning and faster response generation. Qwen3 is trained on a massive and diverse multilingual corpus (about 36 trillion tokens covering 119 languages and dialects), which enhances its cross-lingual understanding and makes it competitive with top open-source models in benchmarks of coding and general capabilities. Qwen3 models undergo multi-stage fine-tuning to improve instruction-following and align outputs with human preferences. This fine-tuning enhances coherence and relevance in generated responses. In our experiments, we fine-tuned the unsloth/Qwen3-8B-bnb-4bit checkpoint^3^.

### Training and evaluation of the language models

**Fig. 1:**
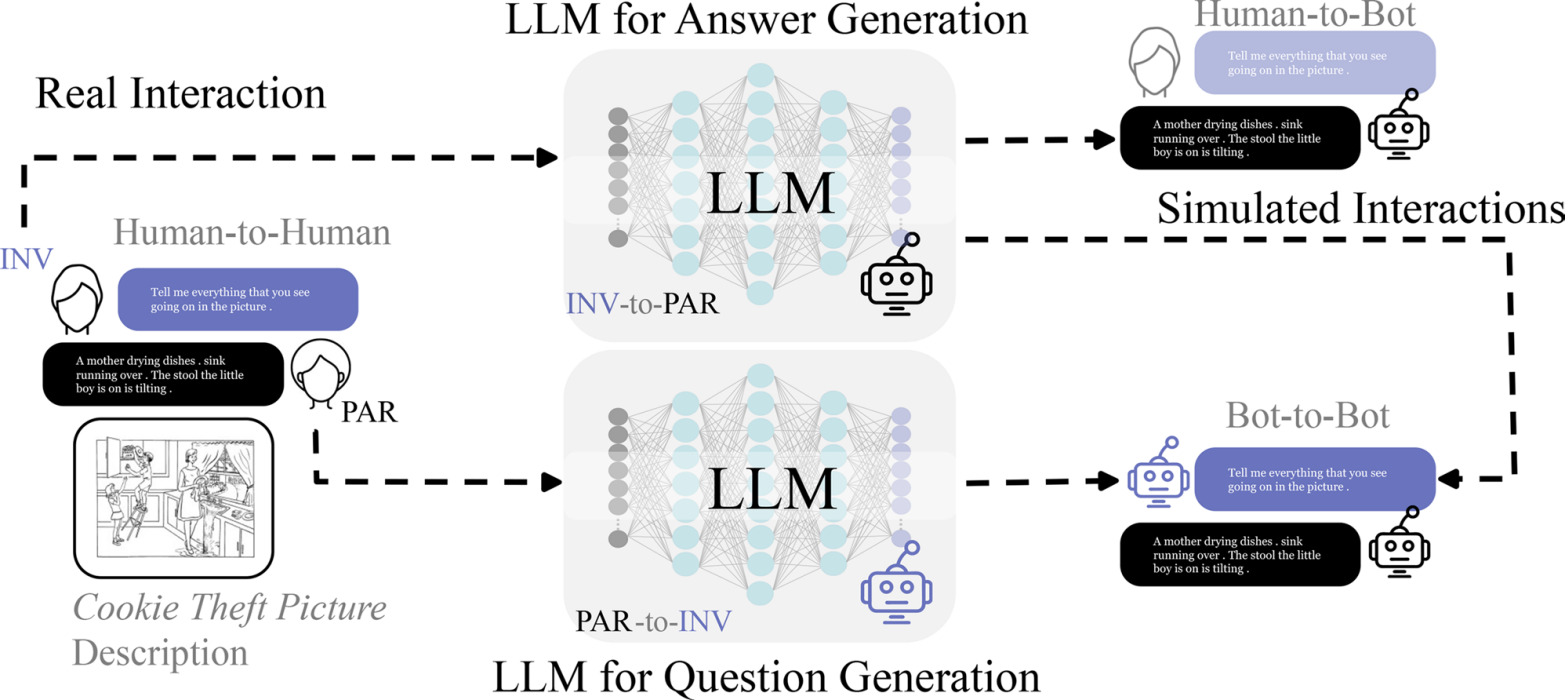
Training and evaluation methodology followed in this study. Two LLMs were fine-tuned for open QA to simulated interactions in cognitive assessments using the *Cookie Theft Picture*. The data is structured as QA pairs from human-to-human interactions. The first LLM (above) is trained for answer generation, using interviewer (INV) questions to elicit participant (PAR) responses. The second LLM is used for question generation, using PAR’s responses to formulate new questions. The models are evaluated in two scenarios: human-to-bot and bot-to-bot

The LLMs in this study were fine-tuned using five-fold Cross-Validation (CV). Each model was trained separately on data from AD and HC groups. Figure 1 summarizes the LLM-based interview generation methodology. The source code will be released on GitHub upon acceptance.

**Model Training:** We focused on training two distinct LLMs tailored to enhance the interactivity and adaptability of our dialogue systems. *Answer Generation LLM* is trained to generate answers, using the INV’s question as input and the PAR’s response as the output. *Question Generation LLM* is trained in reverse; it uses the PAR’s response as the input to generate questions that an INV might ask. To further enhance the dataset, we performed data augmentation through paraphrasing and back-translating (English–German/English–Spanish), i.e., quadrupling our data. We selected these languages because they preserve more of the original meaning while introducing minor phrase changes. Paraphrasing was conducted using a pre-trained T5 model.

**Evaluation Scenarios:** Two communication setups were used for the evaluation. *Human-to-Bot*, where original questions from the dataset were fed directly to the models and their responses were used for analysis. This assesses how effectively the LLM can produce coherent and contextually appropriate answers. *Bot-to-Bot*, involved simulating conversations between two models, one model posing questions and the other providing responses creating a dynamic dialogue loop. This was particularly useful in exploring the potential of data augmentation and assessing interaction dynamics between models.

### Text-based AD detection setup

A Multi-Layer Perceptron (MLP) was used as the classifier for the AD detection task. Optimal hyperparameters were determined via grid search, with hidden layer sizes in {16, 32, 64, 128, 256, 512}, while number of epochs was set to 50 and learning rate to 0.001 using the Adam optimizer. All experiments were validated using a five-fold CV strategy, ensuring that the same data splits were used during both LLM training and classification to prevent any data leakage. The folds were constructed at the participant level, ensuring that all samples from a given participant were assigned to a single fold. This guarantees that no participant appears in both training and test sets, preventing any form of data leakage. In each iteration, four folds were used for training and validation, and the remaining fold was reserved for testing. The LLMs were trained on the same four folds used to train the classifiers, and evaluated on the same held-out fold, ensuring consistent and speaker-independent evaluation across both generation and classification tasks.

## Experiments & results

Our experiments were structured in three parts. First, we examined the ability of different LLMs to generate narratives under *Human-to-Bot* and *Bot-to-Bot* settings. Second, we evaluated how synthetic narratives affect text-based AD detection, measuring classification performance when real and generated data are combined. Finally, we conducted a human evaluation to assess fluency, coherence, and clinical plausibility of the generated narratives.

### Narrative generation

Models were fine-tuned with a batch size of 8 over 20 epochs, using early stopping (patience = 5), a learning rate of 2e-4, and the AdamW optimizer (8-bit quantized for LLaMA, Mistral, and Qwen), with a weight decay of 0.01 to prevent overfitting. All experiments were conducted using a fixed random seed of 42. Notice that for LLaMA, Mistral and Qwen, we integrated LoRA [32], reducing the trainable parameter count to $\approx$**42 M**. We used publicly available pretrained models (e.g., T5, GPT-2, LLaMA, Mistral) under their respective licenses. The proposed framework was implemented in PyTorch using Hugging Face pipelines. Model training was conducted on a NVIDIA H100 GPU.

**Fig. 2:**
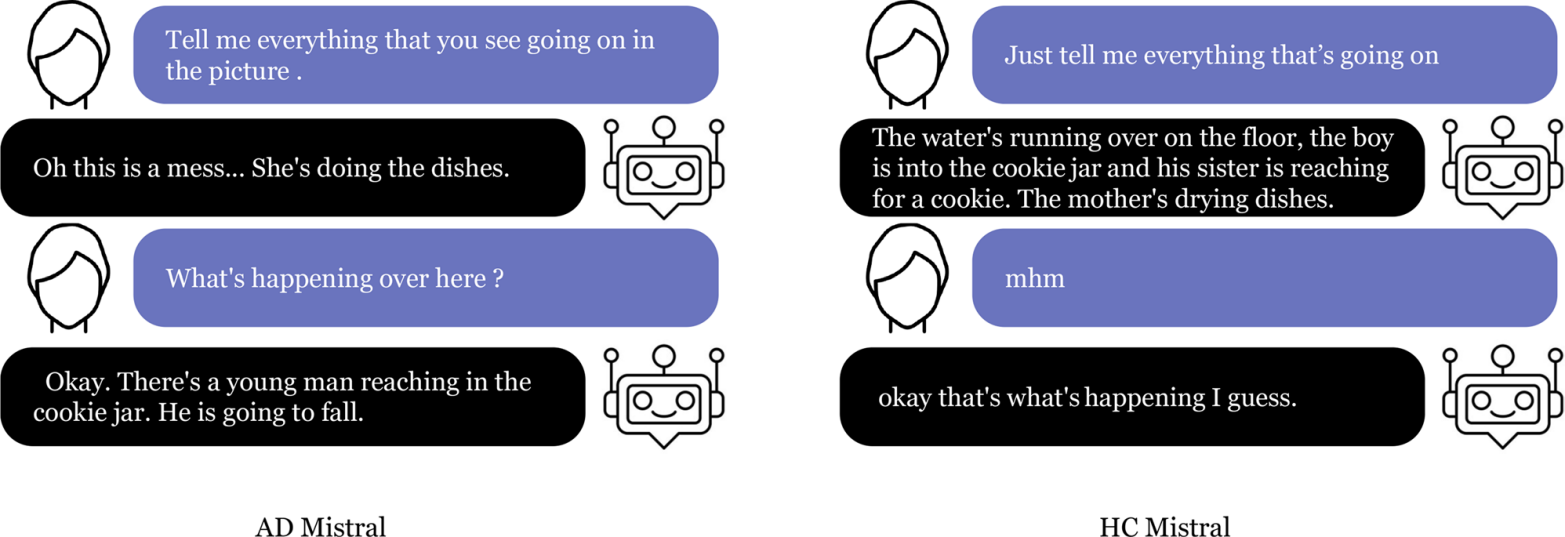
Examples of generated human-to-bot interactions using the Mistral model for an AD patient (left) and a healthy control (right). For illustration purposes, the number of generated characters was limited to a maximum of 120

Figure 2 presents examples from the generated narrative in the Human-to-Bot (H2B) scenario, in which a corpus question is used and the LLM (Mistral model) generates the response to simulate an answer that might be given by a human participant, in this case a patient with AD and HC subject.

Given the open-ended nature of our QA task where the same question might elicit different responses from PARs, we focused on metrics assessing semantic and lexical qualities rather than exact matches. We used a variety of metrics including BLEU, which measures n-gram precision; BERTScore (BScore) and Semantic Score (SemScore), for assessing semantic similarity and accuracy. In the context of generative Question Answering (QA), BLEU scores greater than 0.30 generally indicate acceptable quality. However, this metric is known to have limited correlation with human judgments in open-ended QA tasks [33]. BERTScore, which better captures semantic similarity, is considered strong when exceeding 0.90, with values in the 0.87–0.89 range reflecting reasonable response fidelity. These thresholds are informed by prior QA evaluation work [34].

Tables 4 and 5 provide a comparative analysis of the performance of the LLMs in this study across the described communication scenarios.

**Table 4:** Performance comparison of the evaluated LLMs in the human-to-bot scenario. Models are evaluated using BLEU, SemScore, and BERTScore metrics, reported separately for responses generated under AD and HC conditions. Bold values indicate the highest performance for each metric

Model	Size	BLEU		SemScore		BScore	
		AD	HC	AD	HC	AD	HC
	Small	0.119	0.192	0.670	0.768	0.828	0.839
	Medium	0.194	**0.671**	0.769	0.828	0.834	0.842
GPT-2	Large	0.193	0.670	0.768	0.828	0.833	0.840
	Small	0.035	0.043	0.478	0.515	0.828	0.826
	Base	0.044	0.482	0.512	0.828	0.827	0.838
T5	Large	0.046	0.481	0.518	0.828	0.827	0.834
	Small	0.068	0.123	0.563	0.566	0.841	0.838
	Base	0.114	0.426	0.555	0.845	0.838	0.839
Flan-T5	Large	0.117	0.423	0.561	0.845	0.838	0.841
LLaMA	7B	0.324	0.354	0.740	0.794	0.888	0.885
Mistral	7B	**0.440**	0.379	**0.773**	**0.862**	**0.909**	**0.933**
Qwen	8B	0.335	0.323	0.766	0.784	0.895	0.929

**Table 5:** Performance comparison of the evaluated LLMs in the Bot-to-Bot scenario. Models are assessed on BLEU, SemScore, and BERTScore metrics, reported separately for narratives generated in the AD and HC conditions. Bold values indicate the best performance for each metric

Model	Size	BLEU		SemScore		BScore	
		AD	HC	AD	HC	AD	HC
	Small	0.124	0.207	0.673	0.815	0.827	0.839
	Medium	0.123	0.213	0.674	0.809	0.829	0.842
GPT-2	Large	0.118	0.210	0.678	0.807	0.831	0.840
	Small	0.033	0.041	0.447	0.459	0.832	0.834
	Base	0.032	0.036	0.441	0.456	0.834	0.838
T5	Large	0.029	0.041	0.444	0.467	0.834	0.834
	Small	0.029	0.079	0.413	0.564	0.844	0.839
	Base	0.031	0.075	0.420	0.551	0.845	0.839
Flan-T5	Large	0.063	0.073	0.546	0.548	0.848	0.841
LLaMA	7B	0.324	0.354	0.740	0.814	0.888	0.885
Mistral	7B	**0.379**	**0.438**	**0.773**	**0.842**	**0.908**	0.913
Qwen	8B	0.280	0.303	0.786	0.802	0.895	**0.924**

Model responses are evaluated against specific questions from the original dataset (test sets) to measure the accuracy and relevance of the generated text (H2B). In this scenario, detailed in Table 4, the performance of various LLMs shows considerable variation. GPT-2 maintains consistent BLEU scores across different model sizes, with better semantic performance indicated for HC subjects. The T5 models score lower in BLEU and revealing a notable semantic performance gap favoring HC. The Flan-T5 models demonstrate a gradual improvement in both BLEU and BERTScores, with the best results in the largest model size, especially for HC. LLaMA and Mistral models outperform the other LLMs, with Mistral achieving the highest scores (BERTScores$<$0.9).

Regarding the Bot-to-Bot (B2B) scenario (see Table 5), the GPT-2 model trained on AD patients exhibits a decrease in BLEU scores with increasing model size but shows improved BERTScore and SemScore, suggesting better semantic processing in larger models. However, GPT-2 tends to overfit to short phrases from the patient narratives, frequently reproducing or closely paraphrasing segments of the training data rather than generating more diverse responses that reflect the broader stylistic patterns of the patients’ narratives. Comparable trends can be observed in the T5/Flan-T5 models, with enhancements in BERTScore and SemScore as the model size increases. Similarly to the H2B scenario, the LLaMA, Mistral, and Qwen models report the highest performance across all metrics. An important observation is that some of the newer large language models (e.g., Mistral, LLaMA, and Qwen) tend to generate narratives that resemble those produced by HC subjects. This behavior may occur because the models implicitly correct or adapt the generated content to align with their prior knowledge acquired during pretraining, even after fine-tuning [35, 36].

### Text-based AD detection

The augmented dataset, consisting of original and LLM-generated narratives, was used to train a multilayer perceptron classifier in a five-fold CV setup, to discriminate AD. The input features to the classifier are the average BERT [21] embeddings from google-bert/bert-base-uncased. F1-Scores (F1) are shown in Table 6. Different classification scenarios were established to verify the models’ effectiveness in mimicking the narrative styles of AD/HC subjects. The reported Avg $\uparrow$ DA reflects the impact of data augmentation applied during LLM fine-tuning (Sect. 2.3) to expand the training data. This augmentation influences the generated narratives, which are subsequently used to train the downstream classifier.

**Table 6:** F1-scores for AD classification using generated narratives across different training and evaluation scenarios. Bold values indicate the highest score in each evaluation setting. Value are expressed as mean (standard deviation)

Model	Size	H2B Gen2GT	H2B Mix2GT	B2B Gen2GT	B2B Mix2GT	Avg $\uparrow$ DA
GPT-2	Small	0.567 (0.051)	0.746 (0.105)	0.497 (0.203)	0.550 (0.282)	40%
	Medium	0.551 (0.019)	0.732 (0.075)	0.694 (0.118)	0.563 (0.075)	4%
	Large	0.679 (0.044)	0.759 (0.027)	0.505 (0.133)	0.587 (0.256)	3%
T5	Small	0.523 (0.076)	0.710 (0.018)	0.688 (0.130)	0.354 (0.418)	26%
	Base	0.683 (0.107)	0.713 (0.153)	0.708 (0.124)	0.426 (0.174)	14%
	Large	0.689 (0.123)	0.716 (0.142)	0.674 (0.156)	0.596 (0.107)	10%
Flan-T5	Small	0.689 (0.151)	0.741 (0.134)	0.581 (0.009)	0.651 (0.076)	26%
	Base	0.698 (0.102)	0.756 (0.024)	0.647 (0.012)	0.667 (0.118)	14%
	Large	0.716 (0.152)	0.772 (0.020)	0.709 (0.122)	0.741 (0.021)	10%
LLaMA	7B	0.714 (0.014)	0.756 (0.027)	**0.753 (0.014)**	0.735 (0.079)	12%
Mistral	7B	**0.772 (0.019)**	**0.843 (0.056)**	0.708 (0.098)	0.713 (0.088)	14%
Qwen	8B	0.741 (0.014)	0.812 (0.019)	0.730 (0.082)	**0.741 (0.014)**	9%

Gen2GT: training on generated/testing on original data (GT). Mix2GT: training on a mix of GT and generated text and testing on the GT. Avg $\uparrow$ DA: Average % gain from training with data augmentation

For downstream classification experiments, synthetic narratives were generated to match the size of the original dataset within each fold. This ensures a one-to-one augmentation setup when combining real and generated data (Mix2GT), allowing fair comparison across conditions. Classification only using original data reported 0.72 (std = 0.142) of F1. In the H2B scenario, especially in the Mix2GT setup where models are trained on a mix of original and generated data and tested on original data, models like Mistral excel (F1 = 0.84). In Gen2GT, where models are trained on generated text and tested on original data, simpler models like GPT variants show lower performance ($\approx$0.50). In contrast, models such as LLaMA, Mistral, and Qwen exhibit higher adaptability, scoring up to 0.74 and 0.77, respectively.

B2B scenarios show a modest performance decline, suggesting that models are slightly less effective when the interaction does not involve direct human input. Additionally, a comparison of the different models and simulation scenarios based on AUC scores is provided in the Supplementary Material.

### Qualitative evaluation

Table 7 presents representative narrative responses generated by the evaluated language models for the *Cookie Theft* picture description task under the AD and HC conditions. The examples illustrate qualitative differences in the responses produced by the models and highlight linguistic patterns commonly associated with AD speech. The examples shown were selected based on semantic similarity scores computed from BERT embeddings. This selection strategy allows us to highlight both the similarities and the qualitative differences between the narratives generated by the evaluated language models.

**Table 7:** Representative narratives generated by different language models for the *Cookie Theft* picture description task. Each example corresponds to the model’s response to the prompt “what action do you see going on in that picture?”

Model	AD Narrative	HC Narrative
**GPT-2**		
Small	Boy taking cookies … stool tipping … water running	The boy stands on a stool taking cookies while the girl reaches and the mother dries dishes
Medium	Boy getting cookies from jar, maybe falling from stool	A boy is taking cookies from the jar as his sister reaches up and the sink is overflowing
Large	Mother at sink, water running over, boy on stool taking cookies	The boy is reaching for cookies on a stool while his sister watches and the mother washes dishes
**T5**		
Small	Boy getting cookies	A boy stands on a stool taking cookies while a girl reaches up
Base	Boy taking cookies from jar	The boy takes cookies from the jar while the girl reaches and the sink overflows
Large	Boy on stool taking cookies	A boy is standing on a stool reaching for cookies while his sister watches and the mother is at the sink
**Flan-T5**		
Small	He might fall	The boy is taking cookies while standing on a stool and the girl reaches up
Base	Uh … boy taking cookies	The boy takes cookies from the jar, the stool is tipping and the girl is reaching
Large	Boy taking cookies from jar	The boy is reaching into the cookie jar while the girl reaches up and water spills from the sink
**Recent LLMs**		
LLaMA	Boy reaching into cookie jar, stool tilting, sink overflowing	A boy stands on a stool reaching for cookies while his sister reaches up and the sink is overflowing as the mother dries dishes
Mistral	The boy is taking cookies and the stool may fall while water runs from the sink	The boy is standing on a stool reaching for cookies while his sister reaches up and the sink is overflowing as the mother dries dishes
Qwen	Boy reaching for cookies on stool, water spilling from sink	The boy stands on a stool taking cookies while the girl reaches up and the mother washes dishes as water runs from the sink

Across most models, narratives generated under the AD condition tend to be shorter, less detailed, and often focus on a limited subset of the visual elements in the scene. In contrast, HC narratives typically provide richer descriptions that integrate multiple events and objects in the picture, such as the boy reaching for cookies, the stool tipping, and the sink overflowing. These differences reflect the linguistic characteristics reported in the Pitt Corpus, where speech from individuals with AD often exhibits reduced informativeness and simpler sentence structures compared to healthy speakers.

Model capacity also influences the coherence and informativeness of the generated narratives. Larger and more recent models such as LLaMA, Mistral, and Qwen tend to produce more fluent and contextually complete descriptions, frequently capturing multiple actions in the scene. In contrast, smaller models or earlier architectures such as T5 and GPT-2 often generate shorter or less informative responses.

To quantitatively assess lexical variability and redundancy in the generated responses, we compute intra-dataset similarity using average Jaccard similarity over 3-grams (see Table 8). This metric measures the extent to which responses within the same dataset share common multi-word patterns, where higher values indicate greater repetition and lower variability. As shown in Table 8, ground-truth narratives exhibit very low intra-dataset similarity, reflecting high lexical variability across speakers. In contrast, GPT-2 and T5 show substantially higher similarity values, indicating that generated responses are significantly more repetitive and share common multi-word patterns across samples.

**Table 8:** Intra-dataset similarity measured as average Jaccard similarity over 3-grams. Lower values indicate higher variability

Condition	Ground Truth	GPT-2	T5	Flan-T5	LLaMA	Mistral	Qwen
AD	0.005	0.252	0.034	0.144	0.019	0.004	0.045
HC	0.011	0.152	0.039	0.123	0.037	0.010	0.028

These observations are consistent with the automatic evaluation results reported in Tables 4 and 5, where the more recent models achieved higher semantic similarity scores and overall performance.

Overall, the examples demonstrate that modern large language models can generate narratives that resemble clinically relevant speech patterns observed in both AD patients and healthy controls, supporting their use for synthetic data generation and data augmentation in Alzheimer’s disease detection tasks.

### Human evaluation

**Fig. 3:**
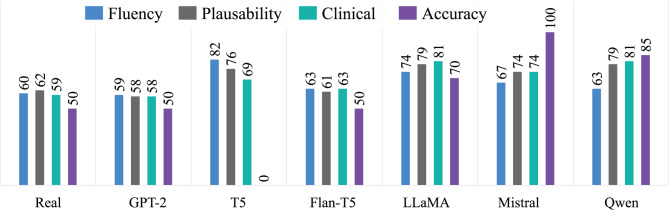
Human evaluation average scores by model

We conducted a human evaluation of generated and real *Cookie Theft Picture* responses using a panel of 18 domain experts to evaluate 20 randomly selected interactions (see Fig. 3). To ensure consistency and robustness of the evaluation, the same annotation protocol was applied uniformly across all models. Interactions were sampled independently per model, maintaining a balanced representation, and the evaluation was conducted under identical conditions for all samples. The randomization procedures was designed to minimize potential bias, and no information regarding model identity or diagnostic labels was disclosed to the annotators at any stage.

For additional details on the human evaluation protocol and annotator profiles, please refer to the Supplementary Material.

They rated each response on fluency, plausibility, and clinical appropriateness using a 5-point Likert scale that was converted into percentage. Additionally, they were asked to judge whether the speaker was more likely to be an AD or a HC. This binary decision was used to compute the diagnostic accuracy of each model’s outputs. Mistral outperformed all other models, which includes real samples, achieving the highest average scores and high diagnostic accuracy. However, due to the small number of human evaluators and samples seen in the survey, these results should be interpreted with caution. While T5 achieved the highest fluency, it scored lowest in clinical appropriateness. Real samples received lower-than-expected scores in fluency and plausibility, likely due to the natural complexity and disfluency of authentic patient speech when judged in isolation. Notably, one of the phoniatricians, who frequently assess AD patients, correctly classified all real interactions, highlighting the value of clinical expertise in interpreting cognitively impaired language.

To better understand the relationship between human-judged quality and downstream task performance, we computed Pearson correlations between human evaluation scores and AD classification outcomes across all generation settings. We first analyzed the relationship between human ratings and automatic generation metrics, as shown in Table 9. Correlations were consistently stronger for HC samples across all metrics. For example, plausibility aligned closely with BLEU and BERTScore in HC responses (*r* = 0.974 and *r* = 0.966, respectively), but showed weaker alignment in AD responses (*r* = 0.342 and *r* = 0.381). This suggests that standard metrics capture quality more reliably in fluent, well-structured language, while AD-like speech, which is often disfluent or fragmented, is less well represented. We then examined how human-evaluated quality related to downstream classification performance.

**Table 9:** Pearson correlation between human evaluation scores and automatic generation metrics. Correlations underlined were not statistically significant. Correlations in bold are both statistically significant and considered strong

Variable	BLEU		SemScore		BScore	
	AD	HC	AD	HC	AD	HC
Fluency	0.234	0.605	-0.052	0.342	0.350	**0.704**
Plausability	0.342	**0.974**	0.270	0.765	0.381	**0.966**
Clinical	0.667	0.324	0.347	0.146	0.737	0.455
Accuracy	0.630	**0.908**	0.713	0.629	0.483	**0.974**

Diagnostic accuracy, as judged by annotators, showed the strongest correlation with classifier performance (see Table 10), especially in the H2B Mix2GT setting (*r* = 0.802). These results suggest that clinical appropriateness is a reliable proxy for assessing the practical utility of generated responses. In contrast, fluency showed negative correlations in H2B settings, implying that overly polished language may not align with clinically realistic patterns.

**Table 10:** Pearson correlation between human evaluation scores and AD classification performance across different generation scenarios. Underlined values are not statistically significant. Bold values represent strong and statistically significant correlations

Variable	H2B Gen2GT	H2B Mix2GT	B2B Gen2GT	B2B Mix2GT
Fluency	-0.267	−0.426	0.451	−0.571
Plausibility	0.084	0.059	0.504	−0.207
Clinical	0.274	0.219	0.349	0.222
Accuracy	**0.730**	**0.802**	0.085	**0.754**

## Discussion & conclusions

This study explored the potential of LLMs to generate meaningful narrative responses for AD detection, using the *Cookie Theft picture* description task as a proxy for cognitive evaluation. We evaluated five LLMs, namely GPT-2, T5, LLaMA, Mistral and Qwen, in terms of their ability to mimic narrative styles observed between AD patients and HC subjects across two communication settings: *Human-to-Bot*, where the model responds to real interviewer prompts, and *Bot-to-Bot*, where both interviewer and participant roles are simulated by LLMs.

### LLM performance across communication settings

Among all evaluated models, Qwen, Mistral and LLaMA consistently outperformed GPT-2 and T5, especially in the H2B condition. In this setup, the presence of human-authored prompts provided contextual grounding, enabling LLMs to generate more coherent, context-sensitive, and semantically rich responses. This resulted in stronger BLEU, BERTScore, and SemScore values, especially for Mistral, which achieved a BERTScore of up to 0.933 and a classification F1 of 0.843 in the H2B Mix2GT setting.

These results are especially noteworthy considering the absence of model-specific fine-tuning. Prior work, such as Balagopalan et al. [20], achieved comparable classification performance (83%) using fine-tuned BERT models on ADReSS data. Notice, that ADReSS is a reduced version of the Pitt Corpus, which may lead to performance changes.

In contrast, T5 models showed lower performance in both linguistic quality and classification. This may be attributed to their training focus on generalized text-to-text transformation rather than interactive or narrative-specific generation, which limits their capacity for role-consistent and semantically grounded dialogue.

### Challenges in fully synthetic interaction (B2B)

In the B2B scenario, we observed a consistent decline in performance across all models. While Qwen, LLaMA, and Mistral remained comparatively strong, the lack of human grounding led to more semantic drift and reduced context relevance in generated dialogues. This suggests that models still rely heavily on the structure and subtle cues present in human-authored prompts to generate clinically realistic content.

Furthermore, B2B interactions may compound distributional artifacts since both sides of the conversation are artificially generated, amplifying model-specific biases. This points to the need for more robust simulation strategies, potentially involving hybrid pipelines that alternate human-generated and machine-generated turns, this to maintain interaction realism.

### Human evaluation and validity

Human evaluations further support the performance metrics, particularly favoring Mistral for fluency, plausibility, and diagnostic accuracy. Interestingly, even real responses received lower scores in fluency and plausibility due to natural disfluencies, underscoring the challenge of evaluating cognitively impaired language outside of context. Clinical appropriateness emerged as the most reliable indicator of downstream classification utility, with diagnostic accuracy showing strong positive correlations with F1 scores in Mix2GT setups ($\textrm{r} = 0.902$).

These findings suggest that future evaluation frameworks should emphasize domain-specific relevance rather than surface-level fluency, especially in clinical applications where linguistic anomalies are diagnostically significant.

### Summary, implications, and future directions

The findings of this study highlight several important implications for both research and clinical applications. The evaluated LLMs (GPT-2, T5, LLaMA, Mistral, and Qwen) were fine-tuned to generate narrative responses in the context of the *Cookie Theft Picture* task, simulating narratives of AD patients and HC subjects. However, the AD classification task relied on a MLP trained on frozen BERT embeddings, without task-specific fine-tuning. This separation underscores the strength of the generated data in enhancing classifier performance without adapting the feature extraction (BERT) model itself. It is important to note that the AD classification component relies on a relatively simple architecture. This design choice was intentional to isolate the impact of synthetic data augmentation rather than optimize classifier performance. More advanced approaches, including LLM-based zero-shot or few-shot classification via prompting or in-context learning (e.g., recent instruction-tuned models such as Qwen), may further improve performance and should be explored in future work.

The results demonstrated that fine-tuned LLMs, particularly Mistral, LLaMA, and Qwen are capable of producing semantically rich and diagnostically useful narratives, especially in the H2B scenario. Their outputs significantly improved classification accuracy when mixed with real data. In contrast, models like GPT-2 and T5 exhibited more variability, often due to their sensitivity to prompt structure and weaker contextual grounding. This suggests that future work should continue to refine prompt engineering strategies and explore instruction tuning techniques to further stabilize generation quality across models.

Another important limitation concerns the potential for memorization and overfitting when fine-tuning language models on relatively small and homogeneous datasets such as the Pitt Corpus. In particular, smaller or less robust models such as GPT-2 occasionally reproduced short phrases or closely paraphrased segments from the patient narratives, suggesting that the model may rely on memorized patterns rather than learning generalized linguistic characteristics of AD speech. This phenomenon has been widely observed in neural language models, which can emit memorized sequences from their training data when prompted with similar contexts [37].

In contrast, larger models such as Mistral, LLaMA, and Qwen showed better semantic generalization and produced more coherent narratives. However, these models may still introduce their own biases derived from large-scale pretraining [35, 36], sometimes generating longer narratives that resemble typical HCs descriptions rather than faithfully reproducing the linguistic variability of AD patients. When deployed in fully synthetic B2B interactions, such tendencies may further amplify stylistic artifacts or distributional biases across dialogue turns. Future work should therefore investigate mechanisms to improve generalization while preserving clinically relevant linguistic patterns, for example through larger and more diverse clinical corpora, controlled data augmentation strategies, and evaluation protocols designed to detect memorization or stylistic drift.

Moreover, given the reliance on English-language data from the Pitt Corpus, extending this framework to multilingual and culturally diverse corpora is a necessary next step. Addressing linguistic variation will help ensure the generalizability of synthetic assessments in global clinical settings. Additionally, as LLMs become increasingly capable of mimicking human-like responses, there is a growing need for robust privacy safeguards. Techniques such as differential privacy, content filtering, and alignment with ethical standards will be essential to prevent the inadvertent generation or exposure of sensitive information. Although this study focuses on the *Cookie Theft* picture description task, the proposed framework is not limited to this specific paradigm. The interaction modeling approach relies on question–answer exchanges between an interviewer and a participant and could be extended to other elicited speech tasks commonly used in neuropsychological assessments, such as narrative recall or semi-structured interviews. Furthermore, because picture description tasks are often used in early cognitive screening, the proposed framework may also support future work targeting earlier stages of cognitive impairment, such as mild cognitive impairment, given appropriate datasets.

Another direction for future research involves improving evaluation methodologies. Current evaluations focus on isolated prompt-response pairs, but future systems should be assessed in the context of full conversations. This would better reflect the dynamic and interactive nature of real-world clinical interviews. Furthermore, the simulation of longitudinal dialogue patterns, e.g., modeling changes over time in a patient’s speech, could provide valuable insights for tracking disease progression.

In summary, this study shows that fine-tuned LLMs can generate high-quality synthetic narratives that effectively augment limited real-world data for AD detection. Even without fine-tuning the classification model, incorporating generated content led to measurable gains in diagnostic performance. Among the models evaluated, Qwen, Mistral, and LLaMA proved most effective, producing fluent, plausible, and clinically aligned responses. While challenges remain in fully synthetic interaction scenarios, the proposed framework offers a scalable approach to data augmentation, laying the groundwork for broader applications in cognitive health assessment.

## Electronic supplementary material

Below is the link to the electronic supplementary material.

Supplementary Material 1

## Data Availability

The DementiaBank Pitt Corpus was used under its academic license terms, and access was granted by TalkBank. For additional details about the corpora used in this study and to request access, please refer to https://dementia.talkbank.org/access/English/Pitt.html.

## Footnotes

1 https://huggingface.co/meta-llama/Llama-2-7b-hf

2 https://huggingface.co/unsloth/mistral-7b-bnb-4bit

3 https://huggingface.co/unsloth/Qwen3-8B-bnb-4bit

## Code availability

The proposed framework was implemented in PyTorch and trained on a NVIDIA H100 GPUs. The source code is available at https://github.com/PauPerezT/generating-ad-narratives.
