# Supplementary material for: Generating Alzheimer’s narratives using large language models
Source: BMC Med Inform Decis Mak. 2026 Jul 7;26:258. doi: 10.1186/s12911-026-03653-4 (PMC13352925; doi:10.1186/s12911-026-03653-4)
Supplement: Supplementary file 1 — Supplementary Material 1 [file 12911_2026_3653_MOESM1_ESM.pdf]

Appendix A Supplementary Material

Figure A1 shows the classification results for all the considered models according to the Area Under the ROC curve (AUC).

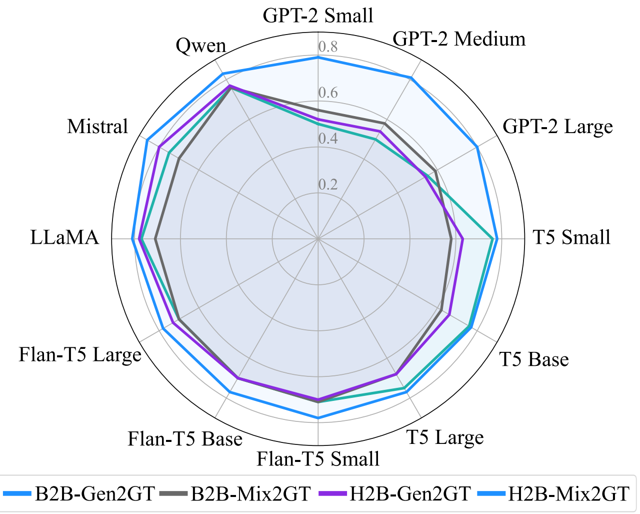

Fig. A1: AUCs for discriminating AD using generated narratives. Gen2GT: training on generated/testing on original data (Ground Truth–GT). Mix2GT: training on a mix of GT and generated text and testing on the GT.

A.1 Human Evaluation Protocol and Annotator Profile

We recruited 20 human evaluators to assess a randomly sampled subset of 20 prompt-response pairs (balanced between models and interaction modes). Each response was rated on three dimensions, fluency, plausibility, and clinical appropriateness, on a 5-point Likert scale. Fluency refers to the question: Is the sentence grammatically correct and natural-sounding? Plausibility to: Could this have been written by a human speaker (e.g., a patient in a clinical interview)? Clinical Appropriateness to: Does the content make sense given the task of describing the picture in a cognitive assessment setting? Additional, a fourth binary question asked whether the response was more likely to originate from an Alzheimer's Disease (AD) patient or a Healthy Control (HC).

Evaluators represented a diverse expert pool:

- 2 Phoniatricians (clinical voice and speech disorder specialists)
- 2 Psychologists
- 2 Linguists with expertise in narrative and disordered speech
- 12 Machine Learning specialists familiar with LLMs and text generation

- 7 Speech and Language Processing researchers (some with overlap with Machine Learning specialists familiar with LLMs and text generation)

In addition to professional background, we collected demographic metadata:

- Gender distribution: 7 female, 13 male
- Highest degree obtained: 6 PhDs, 14 MScs
- 15 university-based, 5 hospital or clinical research centers
